# A New Challenge for Radiologists: Radiomics in Breast Cancer

**DOI:** 10.1155/2018/6120703

**Published:** 2018-10-08

**Authors:** Paola Crivelli, Roberta Eufrasia Ledda, Nicola Parascandolo, Alberto Fara, Daniela Soro, Maurizio Conti

**Affiliations:** ^1^Department of Biomedical Sciences, Institute of Radiological Sciences, University of Sassari, Sassari, Italy; ^2^Department of Clinical and Experimental Medicine, Institute of Radiological Sciences, University of Sassari, Sassari, Italy

## Abstract

**Introduction:**

Over the last decade, the field of medical imaging experienced an exponential growth, leading to the development of radiomics, with which innumerable quantitative features are obtained from digital medical images, providing a comprehensive characterization of the tumor. This review aims to assess the role of this emerging diagnostic tool in breast cancer, focusing on the ability of radiomics to predict malignancy, response to neoadjuvant chemotherapy, prognostic factors, molecular subtypes, and risk of recurrence.

**Evidence Acquisition:**

A literature search on PubMed and on Cochrane database websites to retrieve English-written systematic reviews, review articles, meta-analyses, and randomized clinical trials published from August 2013 up to July 2018 was carried out.

**Results:**

Twenty papers (19 retrospective and 1 prospective studies) conducted with different conventional imaging modalities were included.

**Discussion:**

The integration of quantitative information with clinical, histological, and genomic data could enable clinicians to provide personalized treatments for breast cancer patients. Current limitations of a routinely application of radiomics are represented by the limited knowledge of its basics concepts among radiologists and by the lack of efficient and standardized systems of feature extraction and data sharing.

## 1. Introduction

**Figure 1:**
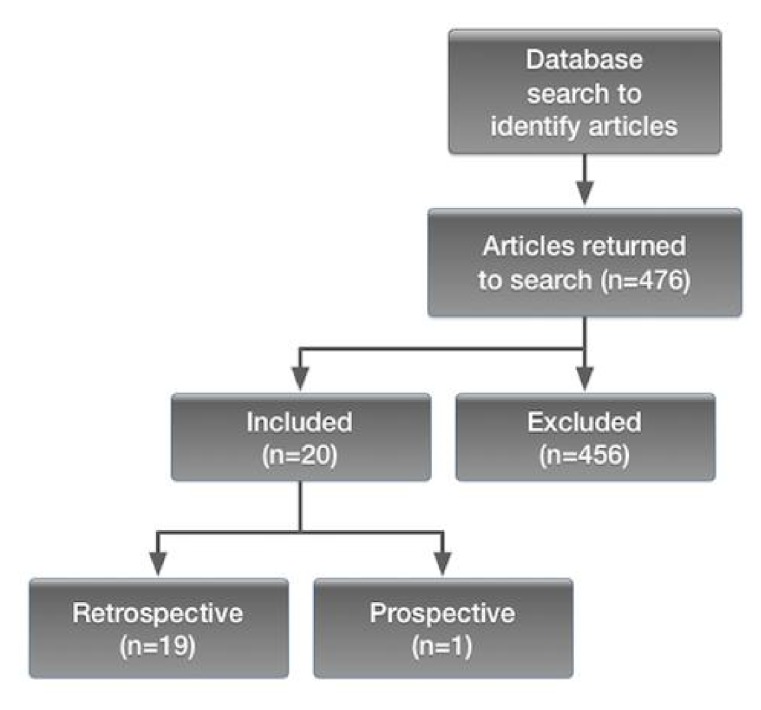


Breast cancer is the most commonly diagnosed cancer and the second leading cause of death for cancer among women worldwide [1]. The prediction of response to treatment and of prognosis is essential in clinical practice in the era of precision medicine [2]. In the past decade, oncologists and radiologists have been showing an increasing interest for the clinical utility of quantitative imaging, encouraged by the significant advancements within the field of medical images analysis. This exponential growth led to the development of radiomics, with which innumerable quantitative features are extracted from digital medical images, usually tomographic, through a high-throughput computing. These features, relating to tumor size, shape, intensity, and texture, provide a comprehensive tumor characterization, defining what it has been called the radiomics signature of the tumor [3]. Radiomics is based on the central hypothesis that extracted quantitative data reflect mechanisms occurring at genetic and molecular levels [4]. Radiomics is a complex process that involves several steps. It begins with acquisition of high-quality images, from which a region of interest (ROI) is identified and segmented either manually or automatically. The ROI can include the whole tumor or some parts of it. Once the segmentation is completed, the selected regions are rendered in three dimensions, becoming volumes. Dedicated software [5–7] then extract quantitative features from the obtained volumes to produce a report, which is inserted into a database and integrated with other data (clinical information, genomic profiles, serum markers, and/or histology data) to be shared across different centers or institutions [3, 8] (Figure 1). A radiomics methodology was first applied to neck and lung cancer imaging [9–11] and more recently to breast imaging [12]. Radiomics seems able to offer imaging biomarkers useful not just to diagnose breast cancer but also to predict treatment response and risk of recurrence. With regard to breast cancer, a radiomics approach has been investigated mainly with Magnetic Resonance Imaging (MRI). However, some studies appearing more recently have explored the potential of radiomics with different imaging modalities: standard mammography, digital breast tomosynthesis (DBT), and ultrasound (US). Aim of this review is to explore the current and potential role of radiomics in breast cancer, focusing on the ability of radiomics to predict malignancy, response to neoadjuvant chemotherapy (NAC), prognostic factors, molecular subtypes, and risk of recurrence.

## 2. Methods and Materials

We referred to PubMed and the Cochrane review database websites to retrieve English-written relevant articles (abstract and/or full-text). Systematic reviews, review articles, meta-analyses, and randomized clinical trials (published from August 2013 up to July 2018) were considered. Keywords typed for our search were as the following: breast cancer and radiomics, breast MRI and radiomics, breast mammography and radiomics, breast tomosynthesis and radiomics, breast ultrasound and radiomics, breast neoplasia and radiomics, breast lesion and radiomics, breast eteroplasia and radiomics, breast MRI and texture analysis, breast MRI and quantitative analysis, breast mammography and texture analysis, breast mammography and quantitative analysis, breast tomosynthesis and texture analysis, breast tomosynthesis and quantitative analysis, breast ultrasound and texture analysis, and breast ultrasound and quantitative analysis. To increase the inclusiveness of our search strategy, we also referred to texts to find other relevant cited manuscripts not retrieved in our initial search. Given the narrative nature of this review, no formal quality assessment was done.

## 3. Results

**Figure 2:**
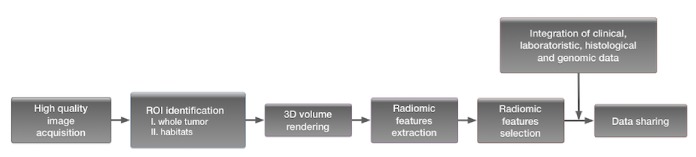


The search on PubMed and on Cochrane databases produced a total of 476 articles; non-English papers, duplicates, case reports, comments, letters, articles that did not considered breast cancer specifically, irrelevant studies, inappropriate data, and comparisons were excluded. All articles presenting quantitative studies but not purely radiomics were excluded as well as those on nuclear medicine imaging. All retro- and prospective original articles that investigated the application of radiomics to breast cancer were included. Twenty papers, 19 retrospective and 1 prospective studies, were selected (Figure 2; Table 1).

## 4. Radiomics and Malignancy

Several studies have investigated the usefulness and reliability of radiomics to discriminate benign breast lesions from cancers, demonstrating that its application might improve the radiologist confidence in the challenging diagnostic task.

### 4.1. MRI

Parekh and Jacobs [13], aiming to find a correlation between radiomics features and different breast tissues of interest, generated radiomics feature maps (RFMs) for visualization and evaluation of radiological images. The radiomics features were then correlated to different breast tissues and compared with quantitative values of radiological parameters. Malignant lesions showed higher values of entropy and the entropy RFM was the most reliable to distinguish malignant from benign lesions, reflecting the tumor heterogeneity and its vascular status. Whitney et al. proposed a radiomics method to investigate whether a set of quantitative features extracted from MR images might help to distinguish luminal A breast cancers from benign breast lesions, compared to using maximum linear size alone [14]. They retrospectively analyzed dynamic contrast-enhanced- (DCE-) MRI of 508 breast lesions and extracted 38 features, which were used to design three different classification protocols. The area under the curve (AUC) for maximum linear size alone was 0.797 in comparison to 0.846 and 0.848 for feature selection protocols including and excluding size features, respectively. Thus, the protocol excluding features related to size was statistically equivalent to that including all features in the ability to distinguish the two pathological entities. The radiomics feature of irregularity was found to play an important role in the feature selection process. In 2017, a retrospective study aimed to establish a potential ability of radiomics to determine the malignant nature of suspicious breast lesions detected on screening X-ray mammography [15]. Supported by emerging evidences on the accuracy of contrast-free breast MRI protocols in the detection of malignant breast lesions [32–34], they employed a radiomics methodology on two contrast-free MRI sequences: Diffusion Weighted Imaging (DWI) and T2-weighted sequences. Two radiomics classifiers allowed distinguishing benign from malignant lesions more accurately (AUC of 0.842-0.851) than the mean apparent diffusion coefficient (ADC) parameter alone (AUC of 0.774), proposed by Bogner et al. with the same scope [35]. However, the inclusion of the mean ADC parameter increased the accuracy of the model, demonstrating the advantages of taking into account previous results and, implicitly, of data sharing. Nevertheless, the performance of the proposed model was lower than that of expert breast radiologists (AUC of 0.959), suggesting that the potential of radiomics in prediction of malignant lesions has to be better assessed. Unenhanced sequences were also used by Bickelhaupt et al., who conducted a multicentric and prospective study to evaluate a radiomics model of suspicious breast lesions (BI-RADS 4 and 5) extracted from breast-tissue-optimized kurtosis MRI by two different vendors to differentiate benign from malignant lesions. The proposed model, evaluated in an independent test set, showed reliable results [16].

### 4.2. US and DBT

A radiomics approach on US imaging and specifically on sonoelastograms was proposed by Zhang in 2017, showing that some sonoelastomic features might help to discriminate between benign and malignant breast tumors [17]. A multicentric and prospective study applied a radiomics approach to DBT for the first time in order to differentiate normal breast tissue from malignant breast tissue in patients with dense breasts [18]. Twenty patients with negative standard mammography who had had a DBT-detected and histology-proven breast cancer were enrolled. Further 20 patients of similar age and breast density with negative DBT and US served as a control group. From 104 radiomics features extracted, 3 (skewness, entropy, and 90 percentile) were found to differ significantly between the two groups. Results also revealed that energy, entropy, and dissimilarity correlated significantly with tumor size and entropy with receptor status too. Despite the small patient sample and the biased selection of features, almost inevitably based on MRI, these preliminary results are encouraging, suggesting that a radiomics analysis of DBT images can be used to facilitate cancer detection and for a better characterization of the detected lesion.

## 5. Radiomics and Neoadjuvant Chemotherapy

NAC, administered before surgery to reduce tumor size and the risk of distant metastases, is often the first line treatment for those patients diagnosed with locally advanced breast cancer [36]. However, less than 50% of patients achieve a pathological complete response (pCR) [37, 38]. A retrospective study published by Braman et al. explored the ability of radiomics to predict pCR to NAC [19], analyzing 99 textural features extracted from the intratumoral and peritumoral regions of T1-weighted contrast-enhanced MRI scans. Authors concluded that radiomics might successfully be employed for the purpose, even more effectively if peritumoral regions are included into the analysis and the receptor status considered.

## 6. Radiomics and Prognostic Factors

### 6.1. Lymph Node Metastases

Determining the axillary lymph node status remains a mandatory requirement of the diagnostic process. In 2017, Dong et al. proposed an optimal multivariable radiomics model able to predict sentinel lymph node (SLN) metastases [20], finding that radiomics features extracted from DWI sequences showed higher correlation with SLN metastases than those extracted from ADC mapping. These results, which certainly need further validation, might help in clinical decision-making with respect to axillary surgery, potentially avoiding invasive procedures in patients at a low risk of SLN metastases.

### 6.2. Peritumoral Fat

Over the decades, numerous studies have demonstrated that obesity is associated with increased incidence and mortality from different forms of cancer, including breast cancer [39, 40]. A retrospective study conducted by Obeid et al. investigated the prognostic impact of peritumoral fat in early breast cancers (T1 and T2 stages) [21]. Authors found a significant linking between a specific peritumoral fat feature, extracted from preoperative MRI sequences and axillary node metastases in patients with body mass index greater than 30. Despite the small sample size of patients, results suggest that a radiomics evaluation of the peritumoral fat might provide valuable noninvasive prognostic data.

### 6.3. Ki67

Ki67 labeling index is routinely used as a prognostic marker in breast cancer patients, to estimate both cell proliferation and therapeutic response [41, 42]. A retrospective study including 377 women diagnosed with invasive breast cancer investigated the possibility of predicting the proliferation marker Ki67 expression through a radiomics approach [22]. Three machine learning schemes were employed to classify cancers in to low- and high-Ki67 expression lesions. Following a semiautomatic segmentation on DCE-MRI, 56 radiomics features (morphological, greyscale statistic, and texture ones) were extracted. Results showed that some of the morphologic features such as perimeter, values of area, and diameter tend to have low values in low-Ki67 tumors, being the high expression of Ki67 associated with a high proliferation rate. Overall, 3 texture features (contrast, entropy, and line likeness) were significantly associated with the Ki67 expression. Liang et al. proposed a new, noninvasive Ki-67 predictor status based on breast MRI [23]. They retrospectively analyzed 318 MRI of breast cancer patients (200 for the training dataset and 118 for the validation dataset), whose Ki67 status was known. Authors selected 30 features and composed a Rad-score for each patient following the analysis of the unenhanced T2-weighted fat suppression sequences and the enhanced T1-weighted. Rad-score calculated on T2-weighted images was significantly associated with Ki67 status, in both training and validation datasets, whereas Rad-score on enhanced T1-weighted did not show correlation with Ki67 expression in the validation cases. These results suggest that a new radiomics marker, obtained with routinely performed unenhanced MRI sequence, might preoperatively predict Ki67 expression in breast cancer.

## 7. Radiomics and Molecular Subtypes

Numerous studies have proposed a radiomics approach to predict breast cancers molecular profile, whose definition is essential to establish the best patient management [43]. Furthermore, the integration between radiomics and genomic features, known as radiogenomics, has revealed promising results in oncology, providing opportunities to better understand tumors behavior and thus to improve diagnosis and prognosis [9, 44].

### 7.1. MRI

In 2015, Guo et al. explored the relationship between radiogenomics features and clinical variables such tumor stage, lymph node metastases and molecular receptor status (estrogen receptor, ER, status; progesterone receptor, PR, status; and human epidermal growth factor receptor-2, HER2, status) [24]. Ninety-one cases of invasive breast carcinomas were included into the analysis. Thirty-eight radiomics features (related to size, shape, morphology, enhancement texture, kinetics, and variance kinetics), extracted from DCE-MRI, were correlated to 144 genomic features for 70 genes (70 gene expression features, 70 copy number features and 4 methylation features). Results showed a significant positive association between all tumor size features and tumor stage, as well as between tumor irregularity and tumor stage, meaning that high-stage tumors tend to be larger and more irregular. Several genomic features were found to be significantly associated with molecular receptor status, whereas no single radiomics feature showed a significant association with ER, PR and/or HER status. Conversely, no isolated genomic features showed a positive correlation with tumor stage and lymph node status. The radiomics feature that correlated the most with the tumor stage was the effective diameter, while the Aurora kinase B gene, AURKB (GE), represented the most useful genomic feature to predict the ER status. However, the model combining radiomics and genomic features showed no higher accuracy in the prediction of invasive breast carcinomas clinical phenotypes in comparison to those considering radiomics and genomic features independently, likely due to the small number of patients enrolled. A retrospective study published in 2016 explored the correlation between quantitative features and cancer receptors status (ER+, ER-, PR+, PR-, HER2+, HER2-, and triple negative, TN) [4]. It was demonstrated that MR image-based tumor phenotypes are significantly associated with receptor status and that heterogeneity is an important feature to discriminate different subtypes, of which, in the near feature, it might be possible to define a radiomics predictive signature that will serve as a virtual biopsy. A set of radiomics features extracted from DCE-MRI was proposed by Wang et al. to distinguish TN breast cancers from other subtypes [25]. Both tumor and its surrounding parenchyma were included in the segmentation for each of the 84 women enrolled. Eighty-five features were extracted and combined with machine learning tools. Five different classification models were designed to differentiate TN cancers against non-TN, ER+, ER-, luminal A, and luminal B cancers. Both accuracy and sensitivity of the proposed models were improved by the inclusion of the background parenchyma quantitative features, whose heterogeneity was found to strongly correlate with TN status. In 2017, Fan et al. investigated the possibility of predicting breast cancers molecular subtypes by using radiomics features extracted from DCE-MRI and integrated with clinical information [26]. They retrospectively analyzed pretreatment breast DCE-MRI of 60 breast cancer patients, where 34 were diagnosed with luminal A breast cancers, 8 with luminal B, 7 with HER2, and 11 with basal-like. Age and menopausal status accounted for the clinical data considered. It was observed that features related to tumor heterogeneity tend to have low values in cancers with best prognosis such as luminal A cancers. Moreover, the clinically aggressive HER2 subtype showed the highest enhancement values, likely due to its raised angiogenesis growth rate.

### 7.2. US and Digital Mammography

In 2018, a radiomics approach based on the extraction of quantitative feature from US images was proposed by Guo et al. to better define the biologic characteristics of invasive ductal carcinoma (IDC) [27]. The analysis included patients with best prognosis IDC (HR+, HER2-) and worst prognosis IDC (TN). Tumor grade was also considered. Radiomics features were sorted into six different categories: shape, margin, boundary, echo pattern, posterior acoustic pattern, and calcification. Low grade HR+, HER- tumors were found to be more irregular in shape, with ill-defined margins, posterior shadowing and hyper- or complex echo. Conversely, high grade TN showed regular shape, a hypo- or complex posterior shadowing and posterior enhancement, similarly to other studies [45, 46]. The echo pattern features were the most effective in the prediction of molecular subtypes. A radiomics approach to be applied on digital mammography with the same aim has been recently proposed by Ma et al. [28]. Thirty-nine features, including morphologic, gray scale statistic, and texture ones, were extracted from the manually segmented area on digital mammography images of 331 invasive breast cancers. A machine learning scheme was employed for the molecular subtypes classifications: triple negative versus nontriple-negative; HER2-enriched versus non-HER2-enriched and luminal versus nonluminal cancers. Four features were significantly associated with tumor subtype, revealing that digital mammography, largely available examination, could provide clinicians with quantitative as well as qualitative information.

## 8. Radiomics and Cancer Recurrence

Li et al. investigated a potential linking between breast cancer MRI phenotypes and multigene assays to predict the risk of recurrence [29]. This retrospective study enrolled 84 patients diagnosed with invasive breast cancers: ductal, lobular, and mixed forms. Thirty-eight computer-extracted images phenotypes were automatic obtained from MRI sequences, describing size, shape, margin morphologic appearance, enhancement texture, kinetic curve assessment, and enhancement-variance kinetics of the cancers. These 38 MRI imaging phenotypes were then correlated with the risk of recurrence scores, calculated for each of the three multigene assays considered: MammaPrint, Oncotype DX, and PAM50, previously developed to predict breast cancer recurrence the former two and the molecular subtypes the latter one. The analysis showed promising results and, accordingly to other studies presented in this review, a combined evaluation of both phenotypic and genomic data might be successfully used to assess the risk of cancer recurrence. A more recent retrospective study proposed a radiomics approach based on preoperative MRI to develop a radiomics signature associated with breast cancer recurrence [30]. They enrolled 294 patients affected by invasive breast cancer appearing as a mass on contrast-enhanced MRI. One hundred and fifty-six features were extracted and grouped into three categories: morphological, histogram-based, and higher-order texture features. A radiomics signature, named Rad-score, was calculated for each patient, who was classified at a high-risk or low risk based on the Rad-score itself. Then, a nomogram including the radiomics signature, MRI, and clinicopathological findings was designed to predict individual cancer recurrence, estimating the disease-free survival (DFS). Results showed higher Rad-scores correlation with worse DFS and that the DFS estimation was more accurate when clinicopathological data were included in the evaluation. Drukker et al. proposed a single new radiomics feature, named most enhancing tumor volume (METV), to be used instead of the functional tumor volume, FTV (a semiautomatically biomarker previously employed for the same purpose) for the prediction of recurrence-free survival [31]. They retrospectively included the same 141 women, affected by invasive breast cancer and treated with NAC, enrolled in the FTV validation dataset. METV, obtained on unenhanced and enhanced MR sequences, performed before and after the first cycle of NAC, was found reliable in the prediction of earlier cancer recurrence, with the advantage of being real-time and automatically calculated.

## 9. Discussion

Radiomics is a relatively new discipline with potentially limitless applications in clinical practice and research [2, 3]. The strengths of this postprocessing tool, however, have been mainly demonstrated in oncology imaging, where radiomics provides a comprehensive noninvasive characterization of the whole tumor, defining what it has been named the radiomics signature of the tumor [3]. Biopsy, which certainly remains central in breast cancer management, cannot be representative of the tumor entirety, whose characterization is mandatory for a thorough understanding of the tumor behavior with respect to treatment response particularly. The studies presented in our narrative review have shown that radiomics is promising in the prediction of malignancy, response to NAC, prognostic factors, molecular subtypes, and risk of recurrence. Results have also suggested that the integration of quantitative information with clinical, histological, and genomic data is key in the era of personalized treatments [3]. However, the application of the proposed radiomics approaches in clinical practice is hampered by the lack of knowledge of its basic concepts among radiologists and by the limited availability of efficient and standardized systems of feature extraction and data sharing. Furthermore, given that the majority of radiomics studies is retrospective and with a relatively small simple size, larger prospective studies are needed to validate these preliminary results.

In conclusion, we believe that the definition of a breast cancer radiomics signature could support clinicians to choose the best treatment option, assigning radiologist a central role in breast cancer management.

## Figures and Tables

**Table 1:** Specifications of radiomics studies included in this narrative review.

**Reference**	**Study design**	**Patients** **(No)**	**Diagnostic modality**	**Radiomics imaging features selected (No)**	**Prediction**	**Sensitivity** (%)	**Specificity** (%)	**Accurancy** (%)	**AUC**
Parekh et al. (2017) [13]	Retrospective	124	MRI (3T)	690 (RFMs)	Malignancy	93	85		

Whitney et al. (2018) [14]	Retrospective	508	MRI (1.5 and 3 T)	38	Malignancy				0.846 (including size features) 0.848 (excluding size features)

Bickelhaupt et al. (2017) [15]	Retrospective	50	MRI (1.5 T)	188	Malignancy				0.842-0.851

Bickelhaupt et al. (2018) [16]	Retrospective	222	MRI (1.5 T)	359	Malignancy	98.4	69.7		

Zhang et al. (2017)[17]	Retrospective	117	US	364	Malignancy	85.7	89.3		

Tagliafico et al. (2018)[18]	Prospective	20	Mammography (DBT)	104	Malignancy				0.567

Braman et al. (2017)[19]	Retrospective	117	MRI (1.5 and 3 T)	99	NAC				0.78 (training dataset) 0.74(independent testing set) 0.83 (HR+, HER2−) 0.93 (TN/HER2+)

Dong et al. (2017)[20]	Retrospective	146	MRI (1.5 T)	25	Prognostic factors				0.847 (training set; model 10 T2-fat suppression) 0.770 (validation set; model 10 T2-fat suppression) 0.847 (training set; model 8 DWI) 0.787 (validation set; model 8) 0.863 (training set; model 10 joint T2-fat suppression/DWI) 0.805 (validation set; model 10 joint T2-fat suppression/DWI)

Obeid et al. (2016) [21]	Retrospective	63	MRI (1.5 and 3 T)	13	Prognostic factors	-	-	-	-

Ma et al. (2018)[22]	Retrospective	377	MRI (3 T)	56	Prognostic factors	77.7	76.9	0.757	0.773

Liang et al. (2018)[23]	Retrospective	318	MRI (1.5 T)	30	Prognostic factors				0.762 (training dataset) 0.740 (validation dataset)

Guo et al. (2015)[24]	Retrospective	91	MRI (1.5 T)	38	Molecular subtypes				0.877 (stage) *∗* 0.693 (lymph node) *∗* 0.789 (ER) *∗* 0.689 (PR) *∗* 0.641 (HER2) *∗*

Li et al. (2016) [4]	Retrospective	91	MRI (1.5 T)	38	Molecular subtypes				0.89 (ER+ vs ER−) 0.69 (PR+ vs PR-) 0.65 (HER”+ vs HER2-) 0.67 TN vs others)

Wang et al. (2015) [25]	Retrospective	84	MRI (3 T)	85	Molecular subtypes	57.0 (TN vs others) *∗∗* 62.0 (TN vs ER+)*∗∗* 53.0 (TN vs PR+)*∗∗* 49.5 (TN vs LumA) *∗∗* 69.5 (TN vs LumB) *∗∗*	94.7(TN vs others) *∗∗* 93.6 (TN vs ER+)*∗∗* 94.1 (TN vs PR+)*∗∗* 89.8(TN vs LumA) *∗∗* 90.0 (TN vs LumB) *∗∗*	90.0 (TN vs others) *∗∗* 89.4 (TN vs ER+)*∗∗* 87.8 (TN vs PR+)*∗∗* 81.8 (TN vs LumA) *∗∗* 84.3 (TN vs LumB) *∗∗*	0.878 (TN vs others) *∗∗* 0.883 (TN vs ER+)*∗∗* 0.859 (TN vs PR+)*∗∗* 0.814 (TN vs LumA) *∗∗* 0.789 (TN vs LumB) *∗∗*

Fan et al. (2017)[26]	Retrospective	60	MRI (1.5 T)	88	Molecular subtypes	88. 2 (LumA) 86.5 (LumB) 81.1 (HER2) 81.1 (basal-like)	76.9 (LumA) 62.5 (LumB) 100 (HER2) 100 (basal-like)		0.867 (LumA) 0.786 (LumB) 0.888 (HER2) 0.923 (basal-like)

Guo et al. (2017)[27]	Retrospective	215	US	463	Molecular subtypes				0.760

Ma et al. (2018)[28]	Retrospective	331	Mammography	39	Molecular subtypes				0.865 (TN vs non TN) 0.784 (HER2 vs non HER2) 0.752 (Lum vs non-Lum)

Li et al. (2016) [29]	Retrospective	84	MRI (1.5 and 3T)	38	Recurrence				0.88 (MammaPrint) 0.76 (Oncotype DX) 0.68 (PAM50 risk of relapse based on subtype) 0.55 (PAM50 risk of relapse based on subtype and proliferation)

Park et al. (2018)[30]	Retrospective	294	MRI (1.5 T)	156	Recurrence	-	-	-	-

Drukker et al. (2018)[31]	Retrospective	162	MRI (1.5 T)	1	Recurrence	-	-	-	-

(i) ^*∗*^AUC considering only radiomics models.

(ii) ^*∗∗*^Considering both tumor and BPE features.

## Abbreviations

ADC: Apparent diffusion coefficient
AUC: Area under the curve
AURKB(GE): Aurora kinase B gene
DBT: Digital breast tomosynthesis
DCE-MRI: Dynamic contrast-enhanced-magnetic resonance imaging
DFS: Disease-free survival
DWI: Diffusion weighted imaging
ER: Estrogen receptor
FTV: Functional tumor volume
HER2: Human epidermal growth factor receptor-2
IDC: Invasive ductal carcinoma
METV: Most enhancing tumor volume
MRI: Magnetic resonance imaging
NAC: Neoadjuvant chemotherapy
pCR: Pathological complete response
PR: Progesterone receptor
RFM: Radiomic feature maps
ROI: Region of interest
SLN: Sentinel lymph node
TN: Triple negative
US: Ultrasound.
